# Complete chloroplast genome of red seaweed *Halymenia maculata* (J. Agardh, 1885)

**DOI:** 10.1080/23802359.2019.1624649

**Published:** 2019-07-11

**Authors:** Wei Tan, Rongxia Wang, Hongtao Liu, Yongbo Wang, Hongji Ke, Jiawei Fan, Fuxiao Chen

**Affiliations:** Hainan Provincial Key Laboratory of Tropical Maricultural Technologies, Hainan Academy of Ocean and Fisheries Sciences, Haikou, China

**Keywords:** Chloroplast genome, *Halymenia maculata*, phylogenetic analysis

## Abstract

In this study, we first assembled and characterized the complete chloroplast genome of red seaweed *Halymenia maculata.* It is 190,431 bp in length, with an AT content of 70.56%. In total, 122 genes were identified, and they consisted of 87 protein-coding genes, 33 tRNA genes, and two rRNA genes. The chloroplast genome of *H. maculata* did not show an obvious quadripartite structure. A total of five microsatellites (SSRs) were identified in the genome using MISA. Phylogenetic analysis revealed that *H. maculata* was first clustered with *Grateloupia filicina* and *Grateloupia taiwanensis* in a monophyletic clade that provides useful data for the phylogeny and taxonomy of Rhodymeniophycidae.

*Halymenia maculata*, sometimes mistaken for red plastic bags, is a macroscopic and leathery red alga which belongs to the family Halymeniaceae. Clumps of this seaweed have an extensive distribution in Indo-Pacific region including Vietnam, Philippines, Singapore, Indonesia, and Hawaii (Abbott 1996; De Smedt et al. 2001; Kawaguchi 2002), mainly seen in Hainan Island in China. It is often attached to coral rubble under the tide. Despite more than sixty species discovered in *Halymenia*, many remain poorly known due to the scarce information available. For a large number of species with high economic and medicinal value have been identified in *Grateloupia* which also belongs to Halymeniaceae (Wang et al. 2007), it is speculated that a potential research value also may be found in some species of *Halymenia*.

The fresh thallus of *H. maculata* was collected from Wuzhizhou island in Sanya, Hainan province, China (N18°18′53.40″, E109°45′51.95″) for the total DNA extraction, and the dried samples were stored in the Qionghai research base of Hainan Academy of Ocean and Fisheries Sciences for reference. The whole-genome sequencing was conducted with 150 bp pair-end reads on the Illumine Hiseq Platform. The genome was assembled with NOVOPlasty, and the annotations of genome were submitted to GenBank database (Accession Number: MK783267).

The complete chloroplast genome of *H. maculata* was 190,431 bp in length. The content of A, T, G, and C in the genome was 35.80%, 34.76%, 14.44%, and 14.99%, respectively. Besides, the overall AT content of the whole chloroplast genome was 70.56%. It contained 122 genes, including 87 protein-coding genes (PCGs), 33 tRNA genes, and two rRNA genes. Like the inferior seaweeds, the chloroplast genome of *H. maculata* did not show a typical quadripartite structure and lack the large rRNA operon-encoding inverted repeat (IR). 37 PCGs and 22 tRNA genes were encoded on the forward strand, and 50 PCGs, 11 tRNA genes, and two rRNA genes were encoded on the reverse strand. Additionally, a total of 5 microsatellites (SSRs) were identified in the *H. maculata* chloroplast genome using MISA. Among these SSRs, four were mononucleotides (A/T), and one was trinucleotide (AAG)_5_.

A phylogenetic analysis was carried out based on super matrix of 21 PCGs in 22 chloroplast genomes of species in Rhodymeniophycidae using RAxML software with 1000 bootstrap replicates. The result (Figure 1) revealed that *H. maculata* was first clustered with *Grateloupia filicina* and *Grateloupia taiwanensis* in a monophyletic clade. The topology relationships of Rhodymeniophycidae was consistent with the previous work (Wang et al. 2001; Schneider et al. 2010; Tan et al. 2015). The data of *H. maculata* chloroplast genome will provide new evidence for further studies on phylogeny and taxonomy of the red seaweed Rhodymeniophycidae (Lee et al. 2016).

**Figure 1.**
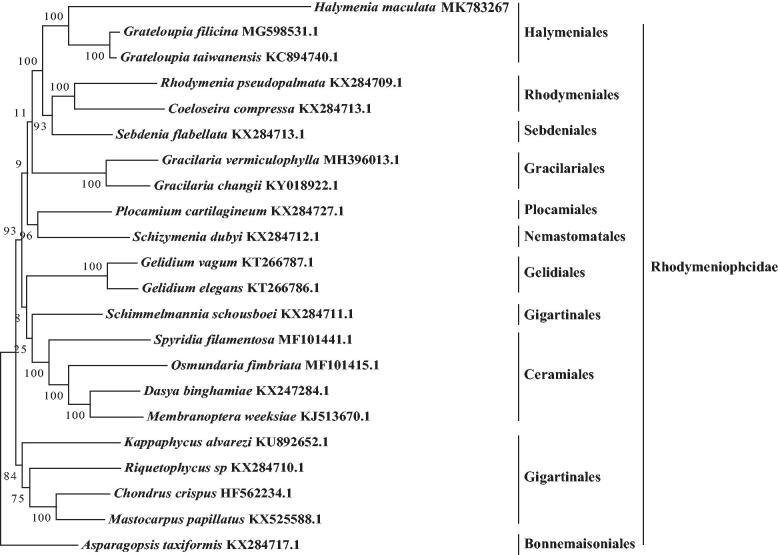
Phylogenetic tree of 22 species based on 21 PCGs from chloroplast genomes using maximum-likelihood (ML) method.
